# Prevalence and associated factors of timely initiation of breastfeeding among mothers at Debre Berhan town, Ethiopia: a cross-sectional study

**DOI:** 10.1186/s13006-016-0086-5

**Published:** 2016-10-03

**Authors:** Getachew Tilahun, Getu Degu, Telake Azale, Askal Tigabu

**Affiliations:** 1 Institute of Medicine and Health Sciences, College of Health Sciences, Department of Public Health, Debre Berhan University, Debre Berhan, Ethiopia; 2 Public Health at College of Medicine and Health Sciences, Institute of Public Health, University of Gondar, Gondar, Ethiopia; 3 College of Medicine and Health Sciences, Institute of Public Health, University of Gondar, Gondar, Ethiopia; 4 Regional Quality Coordinator at Ethiopian Commodity Exchange, Addis Ababa, Ethiopia

**Keywords:** Breastfeeding, Timely initiation, Prevalence, Infant feeding, Optimal breastfeeding

## Abstract

**Background:**

Timely initiation of breastfeeding is defined as putting the newborn to breast within one hour of birth. It serves as the starting point for continuum of care for the newborn health and development. In Ethiopia, there is a considerable variation on timely initiation of breastfeeding among regions. The main aim of this study was to determine prevalence rate and investigate factors associated with timely initiation of breastfeeding practice among mothers in Debre Berhan town, Ethiopia.

**Methods:**

A community based cross-sectional study was conducted at Debre Berhan town from April 1 to 30, 2013. A total of 416 mothers who had given birth within the last six months were selected by using simple random sampling technique. Descriptive statistics, bivariable and multivariable logistic regression analysis were employed to identify factors associated with timely initiation of breastfeeding.

**Results:**

The prevalence rate of timely initiation of breastfeeding was 62.6 %. The odds of timely initiation of breastfeeding was high among mothers who have monthly income of greater than 1969 Ethiopian Birr (ETB) (adjusted odds ratio [AOR] 2.77; 95 % Confidence Interval [CI] 1.21, 6.32). Having extended family (AOR 0.5; 95 % CI 0.27, 0.95), not being counseled about timely initiation of breastfeeding during antenatal care (AOR 0.40; 95 % CI 0.18, 0.88), delivered by cesarean section (AOR 0.11; 95 % CI 0.04, 0.33), delivery attended by traditional birth attendants or relatives (AOR 0.22; 95 % CI 0.05, 0.87), and not feeding colostrum (AOR 0.07; 95 % CI 0.02, 0.23) were negatively associated with timely initiation of breastfeeding.

**Conclusion:**

The practice of timely, also known as early, initiation of breastfeeding was suboptimal. Nearly 40 % of the mothers did not start breastfeeding within one hour after delivery. Findings suggest that in order to improve timely initiation of breastfeeding practice, interventions need to target mothers with extended family, poor socioeconomic status and caesarean delivery. Moreover, mothers who discard colostrum and those who do not deliver under the assistance of health care professional need attention and emphasis has to be given for the breastfeeding counseling service given at antenatal service outlets.

## Background

Breastfeeding provides an ideal food for healthy growth and development of infants and children. Optimal breastfeeding practice includes timely initiation of breastfeeding, exclusive breastfeeding for the first six months and continued breastfeeding up to the age of two years and beyond along with appropriate complementary feeding [1, 2]. Timely initiation of breastfeeding is defined as putting the newborn to breast within one hour of birth [3]. It is one of the ten steps of successful breastfeeding practice and one of the core indicators of assessing appropriate infant and young child feeding practice [4].

In countries where there is high neonatal mortality, infection contributes to almost half of all neonatal deaths. Timely initiation of breastfeeding can help to prevent neonatal deaths caused by infections such as sepsis, pneumonia and diarrhea [5, 6]. The risk of death as a result of infection increases with increasing delay in initiation of breastfeeding after one hour. Late initiation of breastfeeding, after day one for example, was associated with a 2.6-fold increased risk of infection-specific neonatal mortality [7]. Whereas approximately 7.7 % and 19.1 % of all neonatal deaths may be avoided with universal initiation of breastfeeding within the first day and first hour of life respectively [8].

Globally, around four million newborns die, most from preventable causes each year. Death in the neonatal period accounts for 41 % of all deaths in children under five years [5]. Most of these deaths occur during the first seven days of life which is known as the early neonatal period [6].

The risk of newborn death during the first day of life is close to one percent globally, and two third of the burden occurs just in ten countries including Ethiopia [5].

Even though timely initiation of breastfeeding is important for their survival, only 39 % of newborns in developing countries are put to breast within an hour of birth; 47 and 31 % in Africa and Asia respectively [9]. In Africa including Ethiopia, breastfeeding practice is universal where more than 90 % of mother breastfeed, but it is sub optimal [10, 11]. Millennium development goal (MDG) 4 calls for a two- third reduction in death rate for children under five years of age, from 95 per 1000 live births in 1990 to 31 per 1000 live births in 2015. But it sounds impossible to achieve this target without strong effort to reduce neonatal mortality which takes big share of child mortality [12].

In Ethiopia, the problem of malnutrition begins early in life, primarily during the first 12 months due to suboptimal infant feeding practices including late initiation of breastfeeding. It contributes to the country’s high under five year’s child mortality rate, making Ethiopia the sixth country in the world in terms of highest number of under five deaths [1]. Nationally, timely initiation of breastfeeding is 52 % and initiation within one day is 80 %. There is a considerable variation on timely initiation of breastfeeding practice between regions; the highest prevalence was in South Nations Nationalities and People Region (SNNPR) and Dire-Dawa region, 67 and 66 % respectively and the lowest in Amhara region where this study was conducted and Somalia region, 38 and 40 % respectively [11]. Various studies indicate that the prevalence of timely initiation of breastfeeding ranges from 52.4 to 77.9 % at different places across the country [13–15]. This poses a great challenge to the national strategies and programs designed to ensure child nutrition and child survival which are being implemented in the country [1, 16, 17].

Multiple factors interfere with timely initiation of breastfeeding practice in Ethiopia. According to one study in Amhara region, that colostrum discarding and pre-lacteal feeding practices were challenges to timely initiation of breastfeeding [18]. Additionally, maternal education, place of delivery, gestational age at delivery, mode of delivery, type of delivery attendant, prenatal guidance on breastfeeding, postpartum counseling about breastfeeding and parity were reported to be among factors affecting the practice of timely initiation of breastfeeding elsewhere [13–15, 19–26].

Therefore, this study aimed to assess the prevalence and specific factors influencing timely initiation of breastfeeding practice in Debre Berhan town, Ethiopia.

## Methods

### Study setting and design

A community based cross sectional study was conducted in Debre Berhan town from April 1 to 30, 2013. The town is located at a distance of 130 km away to the North-East of Addis Ababa, the capital city of Ethiopia. It is the capital city of North-Shoa administrative zone of Amhara region. It has nine kebeles (smallest administrative units in Ethiopia) consisting of a total population of 81,756; among which 19,214 were mothers of reproductive age groups and 11,037 were children less than five years of which 574 of them were less than six months old.

### Sample size and sampling procedure

Sample size was determined by using single population proportion formula: *n* = z^2^_α/2_ p (1-p)/ d^2^ with assumption of 57 % prevalence (*p*) of timely initiation of breastfeeding in urban setup [11], 95 % confidence level (1.96), 5 % desired precision (d) and 10 % non- respondents. The final sample size was 416 (n) mothers.

A stratified random sampling was employed to select 416 study subjects from the study population. The source population were all mothers who had children less than six months of age and the study population were those mothers who were residents of the town. Mothers who were not permanent residents of the town and seriously ill at the time of data collection were excluded from the study. To select the required sample population, list of mothers with children less than six months of age residing in all kebeles of Debre Berhan town was obtained from the respective kebele’s health extension workers registration book and a sampling frame was constructed first. Then, using stratified random sampling technique and computer generated random numbers, mothers were selected proportionally from all kebeles and included in the study.

### Data collection tools and procedures

Data were collected using structured, pretested and interviewer administered questionnaire which was adopted from Ethiopian Demographic and Health Survey (EDHS) 2011 document [11]. To maintain consistency, the questionnaire was first translated from English to Amharic, the native language of the study area, and was retranslated back to English by professional translators and Public Health experts. Eleven female diploma nurse data collectors and three Health Officer (BSc) supervisors were recruited for the study. Training of data collectors and supervisors was conducted for one day. The tool was pretested on 5 % of the total sample out of the study area. Supervisors and principal investigator checked the completeness, accuracy and consistency of the collected data daily.

### Operational definitions and study variables

- Timely initiation of breastfeeding- refers to if a mother who put her baby to breast within one hour following delivery [3]. It was dichotomized as “yes” and “no” and assigned “1” and “0” respectively for the analysis. To reduce recall bias, we considered only mothers with recent delivery within in the last six months and used important postpartum events which probably end within one hour of after delivery like delivery time of placenta as a probing reference to enable mothers guess the exact time of initiation.
- Type of family- nuclear family refers to a family with married man and woman and their biological children. Whereas extended family refers to a family living with grandparents or uncles or aunts [27].
- Index child- child under age of six month whose mother was our study subject.
- Gestational age at delivery- declared as term or pre term based on a mothers’ perception of gestational age at delivery of index child.
- Prelacteal feeding- refer to any foods and drinks given to a newborn baby before breastfeeding has started [28].
- Previous history of breastfeeding experience – mother having ever breastfed any of her live births prior to the index child.
- Salary was classified based on the tersiles of Ethiopian civil service 18 level salary scale as low (monthly income of less than or equal to 817 ETB), medium (monthly income of 818 to 1968 ETB) and higher (monthly income of greater than or equal to 1969 ETB).

### Data analysis

Data were entered into EPI INFO version 3.5.3 statistical software and exported to Statistical Package for Social Sciences (SPSS) version 20 statistical software for analysis. Descriptive statistics, including frequencies and proportions were used to summarize the variables. Binary logistic regression analysis was used to identify each independent variable’s association with dependent variable. Variables with a *p* - value less than or equal to 0.2 in bivariable analysis were entered in to multivariable logistic regression model for further analysis and analyzed using back ward logistic regression method and a *p*-value of less than 0.05 were considered as significantly associated. The strength of association between dependent and independent variables was expressed by using odds ratio with a 95 % confidence interval.

## Results

### Socio-demographic characteristics

A total of 409 mothers participated in this study with a response rate of 98.3 %. The mean age of mothers was 27.8 (±2.4 SE) years and infants 93.2 (±2.6 SE) days. Majority of the study participants (87.8 %) were from Amhara ethnic group. More than half (58.2 %) of mothers live in an extended family. The monthly income of family was low for one third of mothers (33.3 %) and only less than quarter (21.8 %) of mothers had high monthly income (Table 1).

**Table 1:** Socio demographic characteristics of mothers with children under six months of age at Debre Berhan Town, Ethiopia, April 2013

Characteristics	Number (*n* = 409)	Percent (%)
Age		
15–19	6	1.5
20–24	117	28.6
25–29	147	35.9
30–34	81	19.8
35–39	54	13.2
40–44	4	1
Religion		
Orthodox	316	77.3
Muslim	43	10.5
Protestant	31	7.6
Other^a^	19	4.6
Educational status of mothers		
No formal education	145	35.5
Primary education	99	24.2
Secondary education	83	20.3
Tertiary education	82	20
Educational status of the husband (*n* = 360)		
No formal education	110	30.6
Primary education	74	20.6
Secondary education	82	22.8
Tertiary education	94	26.1
Ethnicity		
Amhara	359	87.8
Oromo	30	7.3
Others	20	4.9
Marital status		
Married	346	84.6
Single	38	9.3
Divorced	8	2
Other^b^	17	4.1
Occupation of mother		
Housewives	176	43
Government employee	64	15.6
Private organization employee	49	12
Private work	100	24.4
Other^c^	20	4.9
Family size		
≤3	129	31.5
>3	280	68.5
Family type		
Nuclear	171	41.8
Extended	238	58.2
Monthly income (ETB)		
Lower	136	33.3
Medium	184	45
Higher	89	21.8

^a^include Catholic, Jehovah, Adventist, ^b^include widowed and separated, ^c^include students, beggars, and daily laborers

### Obstetric and health care service characteristics

Nearly half (52.1 %) of mothers were multipara. The gestational age at delivery of the index child was full term for most (95.4 %) of the respondents. Majority (85.3 %) of mothers had antenatal care follow up at least once for the pregnancy of the index child and more than two third (68.2 %) of them had four and above antenatal visits to health institutions. Similarly, higher proportion of mothers had got counseling regarding practice of timely initiation of breastfeeding during their antenatal care visits (88.7 %), delivered at health institutions (88 %), their delivery was assisted by health professionals (89.7 %), had spontaneous vaginal delivery (92.9 %) and got counseled about timely initiation of breastfeeding practice immediately after delivery (85.1 %) to the index child (Table 2).

**Table 2:** Obstetric and health care service characteristics of mothers with children under six months of age at Debre Berhan Town, Ethiopia, April 2013

Characteristics	Number (*n* = 409)	Percent (%)
Parity		
1	169	47.9
≥2	213	52.1
Gestational age at delivery		
Preterm	14	3.4
Term	390	95.4
Post-term	5	1.2
Antenatal care		
Yes	349	85.3
No	60	14.7
Number of antenatal visits (*n* = 349)		
1	21	6
2–3	90	25.8
≥4	238	68.2
Counseling on timely initiation of breastfeeding during antenatal care (*n* = 349)		
Yes	308	88.7
No	41	11.3
Place of delivery		
Home	49	12
Health institution	360	88
Mode of delivery		
Spontaneous vaginal delivery	380	92.9
Caesarean section	29	7.1
Delivery attendant		
Health professionals	367	89.7
Traditional birth attendants	14	3.4
Family/Friends	28	6.8
Counseling on timely initiation of breastfeeding immediately after delivery		
Yes	348	85.1
No	61	14.9

### Practice of breastfeeding

The overall breastfeeding prevalence rate to the index child in the study subjects was 96.1 % (393); among them, 84.5 % of the mothers fed colostrum to their children and prelacteal feeding was practiced by 16.1 % of mothers (Table 3). Regarding time of breastfeeding initiation, only 256 (62.6 %) of mothers have initiated breastfeeding immediately within one hour following their delivery. Whereas, the rest 153 (37.4 %) of participants did not initiate breastfeeding timely, since about 97 (23.7 %) initiated breastfeeding after one hour up to one day followed by after one day to three days 23 (5.6 %), after three days 17 (4.2 %) and 16 (3.9 %) of participants never initiated breastfeeding until the time of data collection.

**Table 3:** Feeding practice among mothers at Debre Berhan Town, Ethiopia, April 2013

Characteristics	Number (*n* = 409)	Percent (%)
Colostrum feeding (*n* = 393)		
Yes	332	84.5
No	61	15.5
Prelacteal feeding (*n* = 393)		
Yes	66	16.8
No	327	83.2
Past experience of breastfeeding		
Yes	190	46.5
No	219	53.5

### Factors associated with timely initiation of breastfeeding

Many of the independent variables which showed statistical significance with the outcome variable in the bivariable analysis remained significantly and independently associated with the same outcome variable in the multivariable logistic regression analysis. In this regard, the odds of timely initiation of breastfeeding was high among mothers who have higher monthly income than mothers with low monthly income category (AOR 2.77; 95 % CI 1.21, 6.32). Whereas, mothers who have extended family (AOR 0.5; 95 % CI 0.27, 0.95) did not receive counseling about timely initiation of breastfeeding during antenatal care (AOR 0.40; 95 % CI 0.18, 0.88), delivered by caesarean section (AOR 0.11; 95 % CI 0.04, 0.33), delivered by traditional birth attendants or relatives (AOR 0.22; 95 % CI 0.05, 0.87), and not feeding colostrum (AOR 0.07; 95 % CI 0.02, 0.23) were less likely to initiate timely. However, prelacteal feeding, place of delivery, parity, maternal education, gestational age at delivery and antenatal visits were not associated with TBF in this study. The details are given in Table 4.

**Table 4:** Bivariable and multivariable logistic regression output of determinants of timely initiation of breastfeeding practice among mothers at Debre Berhan Town, April 2013

Variables	Timely initiation of breastfeeding		COR (95 % CI)	AOR (95 % CI)	*p* - value
	Yes	No			
Type of family					
Nuclear	119	52	1	1	
Extended	137	101	0.59 (0.391, 0.90)	0.51 (0.27, 0.95)	0.033
Monthly income					
Low	77	59	1	1	
Medium	113	71	1.22 (0.77, 1.91)	1.7 (0.91, 3.11)	0.095
High	66	23	2.20 (1.28, 3.94)	2.77 (1.21, 6.32)	0.015
Occupation of mother					
Housewife	106	70	1	1	
Government employee	45	19	1.56 (085, 2.89)	0.97 (0.41, 2.29)	0.95
Private employee	29	20	0.96 (0.50, 1.83)	0.77 (0.33, 1.80)	0.54
Self-employed	61	39	1.03 (0.63, 1.71)	1.56 (0.74, 3.26)	0.24
Others	15	5	1.98 (0.70, 5.70)	1.94 (0.50, 7.54)	0.34
Gestational age at delivery					
Preterm	6	8	1	1	
Term ^a^	250	145	2.30 (0.78, 6.76)	1.15 (0.25, 5.38)	0.86
Number of antenatal visits					
1	10	11	1	1	
2–3	56	34	1.81 (0.70, 4.72)	1.59 (0.46, 5.46)	0.46
4+	173	65	2.93 (1.19, 7.22)	1.60 (0.48, 5.29)	0.44
Counseling on timely initiation of breastfeeding during antenatal care					
Yes	222	86	1	1	
No	17	24	0.27 (0.14, 0.54)	0.40 (0.18, 0.88)	0.02
Place of delivery					
Home	18	31	1	1	
Health facility	238	122	3.36 (1.81, 6.23)	0.72 (0.91, 5.60)	0.75
Mode of delivery					
Vaginal	250	130	1	1	
Caesarean section	6	23	0.14 (0.05, 0.34)	0.11 (0.04, 0.33)	0.0001
Delivery attendant					
Health professional	241	126	1	1	
Traditional birth attendant/ relatives	15	27	0.29 (0.15, 0.57)	0.22 (0.05, 0.87)	0.031
Counseling on timely initiation of breastfeeding immediately after delivery					
Yes	237	111	1	1	
No	19	42	0.21 (0.12, 0.38)	0.54 (0.16, 1.82)	0.32
Colostrum feeding					
Yes	241	91	1	1	
No	15	46	0.12 (0.07, 0.23)	0.07 (0.02, 0.23)	0.0001
Prelacteal feeding					
Yes	18	48	1	1	
No	238	89	7.13 (3.94, 12.92)	2.35 (0.74, 7.44)	0.15
Breastfeeding experience					
Yes	128	62	1	1	
No	128	91	0.68 (0.45, 1.02)	1.03 (0.59, 1.81)	0.92

*COR* Crude odds ratio, *AOR* Adjusted odds ratio, *CI* Confidence Interval, Significantly associated: *p* value < 0.05

^a^Includes post term deliveries which were 5 in number and all of them were late initiators of breastfeeding

## Discussion

This study assessed the prevalence of timely initiation of breastfeeding and its associated predictors among mothers who have children under six months of age. The findings from this study revealed that nearly two-third (62.6 %) of mothers initiated breastfeeding within one hour of delivery. This indicates that presence of large number of newborns in the study area who were not put to breast timely. The prevalence is higher when compared to the EDHS 2011 finding which was 52, 57 and 38 % for the national, urban area and Amhara region prevalence respectively. In addition to, it is high compared with the study findings in Brazil (47 %), India (23.5 %), Saudi Arabia (11.4 %), Qatar (57 %) and Ethiopia (52.5 %) [11, 13, 19, 22, 24, 25]. The possible explanation for the difference with the EDHS findings could partly be due time horizon and study setting difference as this study is done only on urban mothers. On the other hand the difference in timely initiation of breastfeeding observed between countries could be due to cross cultural difference in breastfeeding practice. Whereas, the prevalence in this study was lower than the findings of studies done in Pulipakkam Village of India and Mekelle town where 97.5 % and 77.9 % of mothers respectively initiated breastfeeding timely [15, 23]. This difference might be due to difference in access to information, socio-economic status and infrastructures.

Mothers with higher monthly income were more likely to initiate breastfeeding as compared to mothers with low monthly income. The finding was supported by similar study [29]. This might be due to mothers with a better monthly income may have access to education, mass medias, health care services and other sources of information that enforce the practice of timely initiation of breastfeeding.

On the other hand, the timely initiation of breastfeeding was lower if the participant lived with extended family. A slightly similar study from Cartagena city of Colombia, reported that mothers with nuclear family breastfeed better than an extended family [30]. This might be due to the presence of grandparents in many extended families in Ethiopian context. These older people are socially respected and acknowledged for dictating the practices that are socio-culturally acceptable by the society, which usually do not sound well scientifically. In addition to this, it might also be due to, extended families usually occur among relatively less educated than educated families, also among poor families than families with a better income. These may limit mother’s access and use of various media of communications and sources of information and even those mothers may give birth at home. This may lead mothers to practice some cultural activities like colostrum discarding and prelacteal feeding (the complementary relationship of prelacteal feeding and delayed breastfeeding initiation has been called a ‘vicious cycle’) prior to the initiation of breastfeeding that in turn delays the initiation time. For example, one study from Ethiopia reported grandmothers and traditional birth attendants as the most influential individuals favoring the practice of colostrum discarding [31] and prelacteal feeding was practiced more by mothers with traditional birth attendant assisted deliveries than with deliveries attended by health professionals and late initiation of breastfeeding was associated with prelacteal feeding [32]. Therefore interventions need to target mothers with extended family structure in order to increase the practice of timely initiation of breastfeeding.

Timely initiation of breastfeeding was low among mothers who delivered by caesarean section. This finding was similar with the study in Brazil, India, Nigeria and Mekelle town [15, 19, 29, 33]. This might be due to the fact that both the newborn and the mothers who deliver by caesarean section are usually stay under various obstetric related health problems, effect of general anesthesia, pain and tiredness. Also health professionals might not give due attention to timely initiation of breastfeeding since they could be occupied by lifesaving activities to the mother as well as to the new born. Similarly, type of delivery assistant was significantly associated with timely initiation of breastfeeding in this study.

Timely initiation of breastfeeding was practiced less likely among mothers whose delivery was attended by traditional birth attendants and families or friends as compared to those mothers whose delivery was attended by health professionals. Studies from Ethiopia reported traditional birth attendants as the most influential individuals favoring the practice of colostrum discarding [31] and prelacteal feeding was practiced more by mothers with traditional birth attendants assisted deliveries than with deliveries attended by health professionals and late initiation of breastfeeding was associated with prelacteal feeding [32]. In addition, studies from Nigeria and Nepal revealed that mothers who deliver in health facilities were more likely to initiate breastfeeding timely than those who deliver at home [29, 34]. This could be since colostum discarding is time taking and prelacteal feeding decreases the newborn’s interest to breast as well as mother’s feeling that her child may feel hungry, it may result in late initiation of breastfeeding among mothers with traditional birth attendants attended delivery. But mothers who deliver at health institutions initiate early and could be due to the health professionals may tend to facilitate timely initiation of breastfeeding. As opposed to this, some studies reported, attendance at delivery by health workers was an important risk factor delaying the initiation of breastfeeding [15], whereas attendance by traditional birth attendants or family and/or friends was reported as a protective factor for early initiation of breastfeeding [26]. The difference might be partly attributed to time horizon, as currently there is better expansion of reproductive health service and information to achieve MDGs related to maternal health and child health that led health professionals to focus on timely initiation of breastfeeding. It implies that it is important to focus on interventions that increasing delivery attended by health professionals in the community. Additionally, it demands guiding traditional birth attendants to give emphasis on timely initiation of breastfeeding as a way out until the institutional delivery becomes culture in the community.

Mothers who were not counseled about timely initiation of breastfeeding during their antenatal visits were less likely to initiate breastfeeding timely as compared to mothers who were counseled. This finding was supported by the study conducted in Brazil and India [24, 33]. This might be due to counseling mothers about the timely initiation of breastfeeding at antenatal clinics enabled mothers to give emphasis on timely initiation of breastfeeding after delivery and led them to practice as compared to those who did not get the service.

In addition, mothers who did not feed colostrum to their child initiated breastfeeding less timely than those mothers who fed colostrum. Similar finding was reported by a study done in Ethiopia where mothers who discard colostrum practiced late initiation of breastfeeding [31]. Delayed initiation could be due to attempt to discard colostrum taking time while milking or pumping it out and even may take two to three days until it is totally removed from the breast and white milk starts to come out therefore results in a delayed initiation of breastfeeding. This indicates that counseling on breastfeeding including the benefits of colostrum feeding should be emphasized in order increase the practice of timely initiation of breastfeeding.

In this study, prelacteal feeding, place of delivery, parity, maternal education, gestational age at delivery and antenatal visits were not associated with timely initiation of breastfeeding. However studies showed that timely initiation of breastfeeding was associated with place of delivery [34], parity [29], maternal education [33, 34] and gestational age at delivery [33]. Therefore studies are needed to further ascertain the relationship of these variables with timely initiation of breastfeeding in the context of the study setting.

### Policy and practice implication

Malnutrition is among public health challenges in Ethiopia and the country is among the top ranking countries in terms of the highest prevalence of various forms of malnutrition [35]. As a result, neonatal, infant and child mortality are also high in the country [11]. However, the implementation of the country’s national strategy for infant and young child feeding adopted baby friendly hospital initiative (BFHI) [1] is not encouraging; only 3 % of hospitals were certified for BFHI [36]. Also findings in this study showed the practice of timely initiation of breastfeeding is suboptimal. Therefore, a substantial increase in timely initiation of breastfeeding practice can be achieved by targeting the national infant and young child feeding (IYCF) intervention on breastfeeding practices towards mothers with extended family, with low socioeconomic status, who do not feed colotrum, who deliver by caesarean section and professionals who provide antenatal care services and traditional birth attendants so as to improve child nutrition and thereby child survival.

### Strength and limitations

The strength of the study is that it is a community based study which enables a minimize selection bias. It also considered only mothers with recent delivery within in the last six months and used important postpartum events which probably end up within one hour after delivery like delivery time of placenta as a probing reference to enable mothers guess the exact time of breastfeeding initiation and reduce recall bias. However the limitation of this study still is, information obtained from mothers could be subject to recall bias. In addition to this the study shares the limitations of cross-sectional studies.

## Conclusion

Improving timely initiation of breastfeeding one of the focus of national strategies and programs aimed at ensuring good nutrition and better child survival which have been strived for [1, 16, 17]. Despite efforts, this study revealed that the prevalence of timely initiation of breastfeeding in the study population was suboptimal as compared to the fact that all children should start breastfeeding timely. Nearly 40 % of mothers in this study did not initiate breastfeeding timely. Not receiving breastfeeding counseling during antenatal care, not feeding colostrum, delivery attended by traditional birth attendants or relatives, and caesarean section delivery were very crucial for timely initiation of breastfeeding practice and need to be intervened. Moreover, mothers with high monthly income are more likely to practice timely initiation of breastfeeding than mothers with low monthly income whereas mothers who have extended families practice timely initiation of breastfeeding less likely than mothers with nuclear family. Therefore interventions need to target those groups in order to increase the practice of timely initiation of breastfeeding.

## Abbreviations

AOR: Adjusted odds ratio
BFHI: Baby friendly hospital initiative
CI: Confidence interval
COR: Crude odds ratio
EDHS: Ethiopian Demographic and Health Survey
ETB: Ethiopian Birr
IYCF: Infant and young child feeding
MDG: Millennium development goal
SE: Standard error
SNNPR: South Nations Nationalities and Peoples Region
SPSS: Statistical Package for Social Science
